# Effectiveness of a Second COVID-19 Vaccine Booster Dose Against Infection, Hospitalization, or Death Among Nursing Home Residents — 19 States, March 29–July 25, 2022

**DOI:** 10.15585/mmwr.mm7139a2

**Published:** 2022-09-30

**Authors:** Kevin W. McConeghy, Elizabeth M. White, Carolyn Blackman, Christopher M. Santostefano, Yoojin Lee, James L. Rudolph, David Canaday, Andrew R. Zullo, John A. Jernigan, Tamara Pilishvili, Vincent Mor, Stefan Gravenstein

**Affiliations:** ^1^Center of Innovation in Long-Term Services and Supports, Veterans Administration Medical Center, Providence, Rhode Island; ^2^Department of Health Services, Policy & Practice, School of Public Health, Brown University, Providence, Rhode Island; ^3^Genesis HealthCare, Kennett Square, Pennsylvania; ^4^Geriatric Research Education and Clinical Center, Louis Stokes Cleveland Department of Veterans Affairs Medical Center, Cleveland, Ohio; ^5^Division of Infectious Diseases and HIV Medicine, Department of Medicine, Case Western Reserve University School of Medicine, Cleveland, Ohio; ^6^National Center for Emerging and Zoonotic Infectious Diseases, CDC; ^7^National Center for Immunization and Respiratory Diseases, CDC ^8^Warren Alpert Medical School, Brown University, Providence, Rhode Island.

Nursing home residents continue to experience significant COVID-19 morbidity and mortality (*1*). On March 29, 2022, the Advisory Committee on Immunization Practices (ACIP) recommended a second mRNA COVID-19 vaccine booster dose for adults aged ≥50 years and all immunocompromised persons who had received a first booster ≥4 months earlier.* On September 1, 2022, ACIP voted to recommend bivalent mRNA COVID-19 vaccine boosters for all persons aged ≥12 years who had completed the primary series using monovalent vaccines ≥2 months earlier (*2*). Data on COVID-19 booster dose vaccine effectiveness (VE) in the nursing home population are limited (*3*). For this analysis, academic, federal, and private partners evaluated routine care data collected from 196 U.S. community nursing homes to estimate VE of a second mRNA COVID-19 vaccine booster dose among nursing home residents who had received 3 previous COVID-19 vaccine doses (2 primary series doses and 1 booster dose). Residents who received second mRNA COVID-19 vaccine booster doses during March 29–June 15, 2022, with follow-up through July 25, 2022, were found to have 60-day VE of 25.8% against SARS-CoV-2 (the virus that causes COVID-19 infection), 73.9% against severe COVID-19 outcomes (a combined endpoint of COVID-19–associated hospitalizations or deaths), and 89.6% against COVID-19–associated deaths alone. During this period, subvariants BA.2 and BA.2.12.1 (March–June 2022), and BA.4 and BA.5 (July 2022) of the B.1.1.529 and BA.2 (Omicron) variant were predominant. These findings suggest that among nursing home residents, second mRNA COVID-19 vaccine booster doses provided additional protection over first booster doses against severe COVID-19 outcomes during a time of emerging Omicron variants. Facilities should continue to ensure that nursing home residents remain up to date with COVID-19 vaccination, including bivalent vaccine booster doses, to prevent severe COVID-19 outcomes.

This analysis emulated target trials that compared the effectiveness of a second mRNA booster dose versus non-receipt among recipients of 2 primary doses followed by 1 booster dose. A series of sequential index dates (i.e., trials) were included to assess VE among nursing home residents during March 29–June 15, 2022, with a maximum of 60-days of follow-up through July 25, 2022. The population included nursing home residents from 196 nursing homes in 19 states^†^ operated by Genesis HealthCare.^§^ Nursing home residents were eligible for study inclusion if they 1) had been present in the nursing home for ≥100 days with <10 days spent out of the facility, 2) had received 3 doses of mRNA COVID-19 vaccine before the index date, and 3) had not received a COVID-19 vaccination in ≥120 days. Nursing home residents were excluded if they had a SARS-CoV-2 infection during the 30 days preceding the index date, had received monoclonal antibodies during the 90 days preceding the index date, or were receiving hospice care. 

Nursing home residents who had been vaccinated on each specific index date were assigned to the treatment group, and those who were unvaccinated but eligible were assigned as controls. Vaccination status was determined using residents’ immunization record from nursing home electronic health record systems. Nursing home residents who had received 3 previous mRNA COVID-19 vaccine doses, irrespective of timing of vaccination were considered to have received the primary series and first booster vaccination. This analysis employed similar analytic methods to other target trial emulations with mRNA COVID-19 vaccines (*4*,*5*). Those who had received the second booster dose were matched to controls exactly by facility of residence and index date with 1:1 nearest neighbor matching with a maximum of 0.2 standardized mean difference in propensity score between pairs. If the matched control subsequently received a second booster dose, follow-up ceased for both the control and matched resident in the treatment group at that time. Propensity scores were estimated using logistic regression adjusting for 1) previous COVID-19 infection history (based on *International Classification of Diseases, Tenth Revision, Clinical Modification* diagnosis code U07.1 or SARS-CoV-2 rapid antigen or reverse transcription–polymerase chain reaction test result), 2) immunosuppressive condition, 3) “do not resuscitate” orders, 4) acute hospitalization during the preceding 90 days, 5) time since last COVID-19 vaccination, 6) length of stay in the nursing home, 7) history of any influenza vaccination during the previous influenza season, 8) age, and 9) number of Charlson index comorbidities (*6*). 

COVID-19 testing followed CDC guidelines for nursing homes, and included testing on admission, readmission, recent exposure, or occurrence of a new symptom. Direct care staff members were tested weekly, and residents could be tested based on recent staff member exposure (*7*). The four outcomes assessed were 1) any incident SARS-CoV-2 infection, defined as a new positive SARS-CoV-2 rapid antigen or reverse transcription–polymerase chain reaction test result, 2) hospitalization for SARS-CoV-2–related illness (transfer to an acute care hospital within 21 days of a new positive SARS-CoV-2 test result), 3) death occurring within 30 days of a new positive SARS-CoV-2 test result, and 4) severe COVID-19 outcomes (combined endpoint of hospitalization or death). Kaplan-Meier estimators were used to estimate VE as 1 − relative ratio of the cumulative incidence curves between groups at each time point. Observations with missing values were excluded from analysis. Sampling with replacement by matched pair with 500 replications was used to generate 95% CIs. Data were collected from nursing home electronic health record systems. Initial data preparation was conducted using SAS software (version 9.4; SAS Institute) and STATA (version 16; Statacorp). All analyses were performed using R statistical software (version 4.0.1; R Foundation). This activity was deemed not to be human subject research by the Brown University institutional review board and was reviewed by CDC and conducted consistent with applicable federal law and CDC policy.^¶^

The analysis included 9,527 unique residents across 196 nursing homes (median of 49 residents per facility [IQR = 35–61]). Among these residents, 9,503 (99.7%) served as controls for ≥1 day of follow-up and 3,245 (34.1%) residents received a second booster dose during the study period and were eligible to be included in the treatment group. In the matched analysis, 1,902 residents were matched 1:1 with controls; 1,343 residents were excluded because they could not be matched to a control. Residents in the matched group had a mean age of 78 years, a median length of stay of 880 days, a median 196 days since the last COVID-19 vaccination, four Charlson comorbidities, and 35.5% were male. Observed characteristics between matched groups were <0.1 standard mean differences (Table 1). Compared with matched residents, the 1,343 excluded residents were similar, with a mean age of 78 years, a median length of stay of 931 days, a median 202 days since the last COVID-19 vaccination, four Charlson comorbidities, and 35% were male.

**TABLE 1:** Baseline resident characteristics of matched second booster dose recipients and first booster dose only controls* — 196 nursing homes, 19 states,^†^ March, 29–July 25, 2022

Characteristic^§^	No. (%)			aSMD
	Total (n = 3,804)	Control* (n = 1,902)	Second booster dose recipients (n = 1,902)	
Male	1,350 (35.5)	663 (34.9)	687 (36.1)	0.03
Black or African American	291 (7.6)	138 (7.3)	153 (8.0)	0.03
Hispanic or Latino	153 (4.0)	86 (4.5)	67 (3.5)	0.05
Serious mental illness or intellectual disability	277 (7.3)	130 (6.8)	147 (7.7)	0.03
Needed language translator	96 (2.5)	54 (2.8)	42 (2.2)	0.04
Current smoker	86 (2.3)	30 (1.6)	56 (2.9)	0.09
Needed dialysis	73 (1.9)	37 (1.9)	36 (1.9)	<0.01
Received influenza vaccination in previous season	2,908 (76.4)	1,418 (74.6)	1,490 (78.3)	0.09
Pulmonary disease	909 (23.9)	436 (22.9)	473 (24.9)	0.05
Diabetes mellitus	553 (14.5)	282 (14.8)	271 (14.2)	0.02
Immunocompromised	524 (13.8)	277 (14.6)	247 (13.0)	0.05
COVID-19 history, ever	2,312 (60.8)	1,143 (60.1)	1,169 (61.5)	0.03
Life expectancy <6 mos	201 (5.3)	106 (5.6)	95 (5.0)	0.03
Do not resuscitate order	1,941 (51.0)	940 (49.4)	1,001 (52.6)	0.06
Any hospitalization, previous 90 days	476 (12.5)	255 (13.4)	221 (11.6)	0.05
Age, yrs, median (IQR)	78 (69–87)	78 (69–87)	78 (69–87)	<0.01
Preindex LOS, days, median (IQR)	880 (511–1,334)	878 (517–1,321)	882 (503–1,345)	<0.01
Time from second dose, days, median (IQR)	196 (182–212)	196 (182–211)	197 (182–213)	0.01
Charlson chronic conditions, median (IQR)^¶^	4 (3–6)	4 (3–6)	4 (3–6)	0.03
No. of COVID-19 tests (14 days), mean (SD)	0.3 (1.1)	0.3 (1.0)	0.3 (1.2)	0.05
No. of COVID-19 tests (90 days), mean (SD)	2.2 (6.0)	2.1 (5.9)	2.3 (6.2)	0.03

**Abbreviations:** aSMD = absolute standardized mean difference; LOS = length of stay; MDS = minimum data set.

* Controls were nursing home residents who had received 3 previous vaccine doses and who were otherwise eligible for receipt of second booster dose but did not receive a vaccination on a given index date during March 29–June 15, 2022.

^†^ Alabama, Arizona, Colorado, Connecticut, Delaware, Kentucky, Maine, Maryland, Massachusetts, New Hampshire, New Jersey, New Mexico, North Carolina, Pennsylvania, Rhode Island, Tennessee, Vermont, Virginia, and West Virginia.

^§^ All information was extracted from nursing home electronic health records; diagnoses are compiled from *International Classification of Diseases, Tenth Revision, Clinical Modification* codes based on Charlson classifications; and other demographic variables are extracted from nursing home MDS assessments (version 3.0), pharmacy, medical orders, or laboratory records. Serious mental illness or intellectual disability refers to item A1500 on the MDS 3.0.

^¶^Total number of Charlson comorbid conditions (e.g., diabetes or congestive heart failure) maximum = 16. A higher number of chronic conditions suggests poor prognosis.

Compared with a first booster dose only, 60-day VE of a second mRNA COVID-19 vaccine booster dose was 25.8% (95% CI = 1.2–44.3) against infection, 60.1% (95% CI = −18.8−91.5) against hospitalization, 89.6% (95% CI = 45.0–100.0) against death, and 73.9% (95% CI = 36.1–92.2) against the severe composite outcome of COVID-19–associated hospitalization or death (Table 2).

**TABLE 2:** Estimated vaccine effectiveness* of a second COVID-19 vaccine booster dose relative to a first booster dose only, for four COVID-19–related outcomes in nursing home residents — 196 nursing homes, 19 states,^†^ March, 29–July 25, 2022

Outcome	Cumulative incidence^§^		Risk difference (per 1,000 residents)	Vaccine effectiveness % (95% CI)**
	Controls^¶ ^(n = 1,902)	Second booster dose recipients (n = 1,902)		
SARS-CoV-2 infection^††^	101	75	−26	25.8 (1.2 to 44.3)
Hospitalization^§§^	9	3	−5	60.1 (−18.8 to 91.5)
Death^¶¶^	8	1	−7	89.6 (45.0 to 100.0)
Severe outcomes***	16	4	−12	73.9 (36.1 to 92.2)

* Through 60 days of follow-up.

**^†^** Alabama, Arizona, Colorado, Connecticut, Delaware, Kentucky, Maine, Maryland, Massachusetts, New Hampshire, New Jersey, New Mexico, North Carolina, Pennsylvania, Rhode Island, Tennessee, Vermont, Virginia, and West Virginia.

^§^ Events per 1,000 nursing home residents.

^¶^ Nursing home residents who received three previous vaccinations and were otherwise eligible to receive a second booster dose but did not receive a vaccination on a given index date during March 29–June 15, 2022.

** Bootstrapped percentile CIs.

^††^ Positive SARS-CoV-2 test result from antigen or reverse transcription–polymerase chain reaction testing.

^§§^ Transfer to acute care hospital within 21 days of a positive SARS-CoV-2 test result.

^¶¶^ Death within 30 days of a positive SARS-CoV-2 test result.

*** Death or hospitalization.

## Discussion

In this analysis, comparing the relative effectiveness of a second booster dose of COVID-19 mRNA vaccines with a single booster dose among eligible nursing home residents in 19 states, VE of a second booster dose against the severe composite outcomes of SARS-CoV-2–associated hospitalization or death was 73.9% and 89.6% for death alone. VE against SARS-CoV-2 infection during a period crossing both Omicron subvariants BA.2 and BA.2.12.1 (March–June 2022) and BA.4 and BA.5 (July 2022) predominance was 25.8%. 

The findings in this report are subject to at least five limitations. First, the point estimates for the findings in the current study are similar to those estimated in previous studies; however, too few hospitalization events were observed to definitely attribute a reduction to vaccination. A recent study from Israel provided similar VE estimates during B.1.617.2 (Delta) variant circulation for a second booster dose (34% against infection, 64% for hospitalization, and 72% against death) in a long-term care setting (*8*). Similarly, a Canadian study reported a 40% relative VE of 4 (versus 3) doses of mRNA COVID-19 vaccine against hospital admission or death among nursing home residents (*9*) and a U.S study reported 80% VE for a second booster dose (compared with no vaccine) against hospitalization among immunocompetent adults aged ≥50 years during Omicron BA.2 and BA.2.12.1 subvariant predominance (*10*). However, comparisons with other published studies are challenging because of differences in methods, population health, and virus characteristics, as well as other factors (e.g., time since the last vaccine dose when VE is measured). Unique features of the present analysis compared with previous studies are the focus on the incremental benefit of the second booster dose compared with 1 booster dose (i.e., 4 versus 3 doses) during a period when Omicron BA.2 and BA.2.12.1 and later BA.4 and BA.5 subvariants were the dominant circulating variants; and use of an emulated target trial design, which applied robust matching to compare persons with similar characteristics at time of vaccination. Second, the composite endpoint of death or hospitalization was included because, in the nursing home population, hospitalizing a resident is subject to many considerations beyond acute illness. The overall health and functional status, life expectancy, resident and family wishes, and general policies of that site are considered. Some residents might have a low likelihood of being hospitalized even with severe COVID-19 illness, which might explain not being able to exclude a null effect for preventing hospitalization alone. Death alone is also problematic because, if residents are hospitalized or transferred, a subsequent death might not be recorded in the nursing home records. Therefore, the composite endpoint of death or hospitalization better described severe outcomes of COVID-19 than did either endpoint alone; however, each outcome was reported separately for interest. Third, the impact of one resident’s vaccination on the effectiveness of vaccination for other residents was not accounted for in this study which might underestimate the direct vaccine effect. Fourth, because of the relatively short follow-up time available for observation (60 days) it was not possible to evaluate potential waning of a second booster dose effect. Finally, the comparison of 4 versus 3 doses might also misclassify some persons who received additional doses because of an immunocompromised status as having received a booster dose.

These results indicate that, compared with a single mRNA COVID-19 vaccine booster dose, a second booster dose provided additional protection against COVID-19–associated severe outcomes among nursing home residents during the Omicron period ending with BA. 4 and BA. 5 dominances. The results support the importance of continued efforts to ensure the nursing home population is up to date on recommended COVID-19 vaccine booster doses including the newly authorized bivalent COVID-19 vaccine.

**Summary**

**What is already known about this topic?**

COVID-19 vaccines have been effective in preventing SARS-CoV-2 infection and associated hospitalizations and deaths among nursing home residents.

**What is added by this report?**

In a large cohort of nursing home residents, receipt of a second mRNA COVID-19 booster dose during circulation of SARS-CoV-2 Omicron subvariants was 74% effective at 60 days against severe COVID-19–related outcomes (including hospitalization or death) and 90% against death alone compared with receipt of a single booster dose.

**What are the implications for public health practice?**

Efforts should be made to ensure that nursing home residents remain up to date with recommended booster doses of COVID-19 vaccines.

## Footnotes

* https://www.cdc.gov/media/releases/2022/s0328-covid-19-boosters.html; https://www.cdc.gov/vaccines/acip/recs/grade/covid-19-second-booster-dose-etr.html (Accessed August 31, 2022).

† Alabama, Arizona, Colorado, Connecticut, Delaware, Kentucky, Maine, Maryland, Massachusetts, New Hampshire, New Jersey, New Mexico, North Carolina, Pennsylvania, Rhode Island, Tennessee, Vermont, Virginia, and West Virginia.

§
https://www.genesishcc.com/

¶ 45 C.F.R. part 46, 21 C.F.R. part 56; 42 U.S.C. Sect. 241(d); 5 U.S.C. Sect. 552a; 44 U.S.C. Sect. 3501 et seq.
